# Usefulness of intravenous immunoglobulin administration to sepsis-induced coagulopathy in ICU patients

**DOI:** 10.1186/cc14027

**Published:** 2014-12-03

**Authors:** N Matsumoto, H Ishikura, Y Nakamura, J Tanaka, M Mizunuma, Y Kawano, S Morimoto, H Kaneyama, A Murai

**Affiliations:** 1 Department of Emergency and Critical Care Medicine, Faculty of Medicine, Fukuoka University, Fukuoka, Japan

## Introduction

Intravenous immunoglobulin (IVIG) has been used as adjuvant therapy for severe sepsis patients expecting the anti-inflammatory effect. However, the effects of IVIG have been controversial. The majority of ill patients with SIRS had coagulation abnormalities. In addition, inflammation and coagulation play pivotal roles in the pathogenesis of sepsis. Moreover, the evidence of extensive cross-talk between these two systems has been increasing. The aim of this study is to investigate the effects of IVIG treatment for inflammation and hemostatic abnormality in sepsis patients.

## Methods

This prospective single-center observational study was conducted in our ICU between January and July 2013. We enrolled 41 patients (≥18 years, admitted to the ICU diagnosed for sepsis, and more than 7 days of ICU stay) and divided them into two groups: IVIG-treated group (IVIG group) and non-IVIG-treated group (non-IVIG group). After that, we compared inflammatory molecule markers (WBC, CRP, procalcitonin (PCT), and interleukin-6 (IL-6)), coagulation/fibrinolysis markers (platelet counts, PT-INR, APTT, D-dimer, TAT, PIC, soluble fibrin (SF), and plasminogen activator inhibitor-1 (PAI-1)), and Japanese Association for Acute Medicine Disseminated Intravascular Coagulation (JAAM DIC) score and positive rate at the admission period (day 1) and days 4 to 7 between two groups. Moreover, the 28-day mortality rate was investigated.

## Results

Nineteen patients were treated with IVIG (IVIG group) and 22 patients were without (non-IVIG group). In the IVIG group, PCT and IL-6 were significantly higher than that in the non-IVIG group at day 1 (*P *< 0.01, and *P *< 0.01). In this group, CRP, PCT, and IL-6 were significantly decreased at days 4 to 7 rather than that at day 1 (*P *< 0.01, *P *< 0.01, *P *< 0.01, respectively). Moreover, the JAAM DIC score was decreased at days 4 to 7 than that at day 1 significantly (*P *< 0.05) in this group. We also confirmed that PT-INR, APTT, TAT, SF, and PAI-1 were significantly improved between day 1 and days 4 to 7. On the other hand, in the non-IVIG group there was significantly decreased IL-6 and TAT only, but not CRP, PCT, PT-INR, APTT, SF, PAI-1 and JAAM DIC score between day 1 and days 4 to 7. For the 28-day mortality rate, the IVIG group was lower than that of the non-IVIG group (IVIG group, 5.3%; non-IVIG group, 18.2%).

## Conclusion

Our study demonstrated that IVIG treatment significantly improved the inflammatory and hemostatic abnormalities in sepsis patients.

